# Association between alcohol consumption and breast cancer incidence and prognosis: A systematic review and meta-analysis

**DOI:** 10.1016/j.breast.2026.104719

**Published:** 2026-02-05

**Authors:** Luca Arecco, Pedro M. Cacilhas, Camila Bobato Lara Gismondi, Marco Bruzzone, Gabriella Gentile, Riccardo Gerosa, Eva Blondeaux, Elisa Agostinetto, Chiara Dauccia, Soraia Lobo-Martins, Rafael Grochot, Kamal S. Saini, Hatem A. Azim, Marcio Debiasi, Alex De Caluwé, Laurence Buisseret, Lucia Del Mastro, Matteo Lambertini, Evandro de Azambuja

**Affiliations:** a Université Libre de Bruxelles (ULB), Hôpital Universitaire de Bruxelles (H.U.B), Institut Jules Bordet, Bruxelles, Belgium; b Department of Internal Medicine and Medical Specialties (DIMI), School of Medicine, University of Genova, Genova, Italy; c Université Paris-Saclay, UVSQ, Gustave Roussy, Inserm, CESP, F-94800, Villejuif, France; d Hospital Israelita Albert Einstein, São Paulo, SP, Brazil; e U.O. Epidemiologia Clinica, IRCCS Azienda Ospedaliera Metropolitana, Genova, Italy; f Department of Radiological, Oncological and Pathological Sciences, Policlinico Umberto I, Sapienza University of Rome, Rome, Italy; g Department of Biomedical Sciences, Humanitas University, Pieve Emanuele, Milan, Italy; h Department of Internal Medicine and Medical Therapy, University of Pavia, Pavia, Italy; i Sarah Cannon Research Institute (SCRI), London, UK; j Addenbrooke's Hospital, Cambridge University Hospitals NHS Foundation Trust, Cambridge, UK; k Cairo Cure Oncology Center, Cairo, Egypt; l Breast Unit, Champalimaud Clinical Centre, Champalimaud Foundation, Lisbon, Portugal; m Department of Medical Oncology, U.O.C. Clinica di Oncologia Medica, IRCCS Azienda Ospedaliera Metropolitana, Genova, Italy

**Keywords:** Alcohol, Breast cancer, Incidence, Recurrences, Prognosis, Public health

## Abstract

**Background:**

While alcohol consumption appears to influence the incidence of breast cancer (BC), its association with prognosis after a BC diagnosis remains less established. This meta-analysis aimed to explore the association between alcohol consumption on both BC incidence and outcomes.

**Methods:**

A systematic literature search was conducted up to May 1st, 2025 (CRD42025593784). Retrospective and prospective studies reporting BC incidence, recurrences, and survival outcomes in women with history of alcohol consumption were included. Analyses according to alcohol intake levels (light, intermediate, heavy consumption) were performed. Main outcomes were BC incidence, BC recurrences, BC-specific survival (BCSS), and overall survival (OS). Pooled relative risk (RR) and hazard ratio (HR) with 95% confidence interval (CI) were calculated.

**Results:**

Out of 5208 screened records, 37 studies including 2,565,920 women were included.

Among 17 studies reporting on BC incidence, any alcohol consumption was associated with an increased BC incidence (RR 1.17, 95%CI 1.09–1.26; p < 0.001). BC incidence increased proportionally with higher levels of alcohol consumption: light RR 1.13 (95%CI 1.05–1.23; p = 0.002), intermediate RR 1.28 (95%CI 1.18–1.39; p < 0.001)​, and heavy consumption RR 1.52 (95%CI 1.38–1.67; p < 0.001).

Among 20 studies assessing BC outcomes, no associations were found between alcohol consumption and BC recurrences (RR 1.02, 95%CI 0.93–1.11) nor BCSS (HR 0.93, 95%CI 0.87–1.00), while light and intermediate alcohol consumption were associated with slightly improved OS: HR 0.85 (95%CI 0.78–0.92; p < 0.001) and HR 0.84 (95%CI 0.75–0.94; p = 0.002), respectively.

**Conclusions:**

Among over 2.5 million women, alcohol consumption was associated with a dose-dependent increased risk of BC, while alcohol consumption did not appear to worsen prognosis in patients with prior BC diagnosis.

## Highlights

• Alcohol consumption is associated with increased breast cancer incidence.
• Breast cancer risk increases with higher levels of alcohol consumption.
• Stronger association observed for hormone receptor-positive breast cancer.
• History of alcohol consumption is not associated with worse prognosis.

## 1 Introduction

Breast cancer (BC) is the most frequently diagnosed cancer among women globally and it represents a major cause of cancer-related deaths [1]. While some risk factors such as sex, age, the presence of pathogenic variants and family history [2,3] are not modifiable, others such as lifestyle habits (i.e., obesity, tobacco, and alcohol consumption) have been identified as modifiable risk factors potentially influencing both BC incidence and prognosis [4,5].

Alcohol intake is a well-established, modifiable risk factor for cancer [6], and its consumption is the third leading preventable cause of cancer in the United States, after tobacco and obesity [5]. Since the late 1980s, alcohol consumption has been linked to an increased risk of at least ten different types of cancers including BC [7]. Biologically, ethanol can induce cancer development through several mechanisms, including its conversion into acetaldehyde (a toxic metabolite that can damage DNA), oxidative stress, and increase in levels of multiple hormones, like oestrogens [8,9]. Recent evaluations have reaffirmed the link between alcohol and BC incidence, estimating that women that consume two alcoholic drinks per day have 10-20% higher risk of developing BC compared to non-drinkers [9]. Consistently, since 1988, the International Agency for Research on Cancer (IARC) classifies alcohol as a Group 1 carcinogen (intended as a carcinogen as there are sufficient evidences of carcinogenicity in humans), alongside tobacco and asbestos [9,10].

While the association between alcohol consumption and BC incidence is based on growing evidence, the impact of alcohol intake on BC prognosis, including recurrences and survival outcomes, remains less clear. Retrospective studies have yielded conflicting results [11], with some reporting no association between alcohol intake and risk of recurrence [12], whereas others suggest that it may negatively affect disease-free survival (DFS) and/or overall survival (OS) [13]. We conducted a systematic review and meta-analysis to comprehensively evaluate the association between alcohol intake and incidence and prognosis of BC in women worldwide.

## 2 Materials and methods

### 2.1 Search strategy and selection criteria

The present systematic review and meta-analysis was conducted according to the Preferred Reporting Items for Systematic Reviews and Meta-Analyses (PRISMA) guidelines [14] and is registered in the PROSPERO database (registration n. CRD42025593784).

A comprehensive literature search was performed up to May 1st, 2025, using PubMed/MEDLINE, Embase, and the Cochrane libraries. The following terms were used: (“breast neoplasms" [MeSH Terms] OR “breast cancer" [Title/Abstract]) AND (alcohol OR “alcohol consumption” OR “ethanol” OR “drinking” OR “alcohol use”) AND (risk OR incidence OR recurrence OR prognosis OR outcome OR mortality OR survival). Only articles published in English were included.

Eligible studies included retrospective, prospective case-control and cohort studies, as well as clinical trials reporting: I) BC incidence in women with a history of alcohol consumption prior to BC diagnosis, compared to non-consumers; II) BC recurrences in patients with a history of BC and alcohol consumption, compared to non-consumers; III) survival outcomes (i.e. BC-specific survival (BCSS) and OS) in patients with a history of BC and alcohol consumption, compared to non-consumers.

The primary outcomes of interest were BC incidence, BC recurrences, BCSS, and OS according to history of alcohol intake.

Studies were excluded if they: I) did not report data for at least one outcome of interest; II) did not allow extraction or estimation of relative risk (RR), odds ratio (OR), or hazard ratio (HR) with 95% confidence intervals (CI); III) were case reports or case series with fewer than 10 patients; IV) were ongoing trials without available results at the time of the search. To better standardize techniques for both BC diagnosis and treatment, studies published before year 2000 were excluded.

The studies retrieved were categorized into two cohorts: cohort 1 included studies reporting on the association between alcohol consumption and BC incidence, cohort 2 included studies reporting on the association between alcohol consumption on BC prognosis in terms of both recurrences and/or survival outcomes. In case a study reported only the data on incidence of contralateral BC in patients with previous history of BC, this study would have been included in cohort 2, considering this as a BC event.

Two reviewers (PC and CBLG) independently screened titles and abstracts. Other two authors (GG and RG) independently participated in the review of the collected papers and in the data extraction. Final discrepancies were resolved through discussion with another reviewer (LA). For each eligible study, the following data were extracted: first author, year of publication, study design and methodology, length of follow-up, number of women included, quantity of alcohol intake, type of alcoholic beverage evaluated in the study, BC incidence and survival outcomes (in terms of recurrences, BCSS, and OS).

To assess whether alcohol intake could influence the type of diagnosed BC subtype, subgroup analyses were conducted according to hormone receptor status (hormone receptor-positive vs hormone receptor-negative disease), menopausal status at diagnosis, and according to the amount of alcohol consumption (categorized as no, light, intermediate, or heavy alcohol intake, when available). For this analysis, varying study cut-offs for alcohol consumption were harmonized into standardized categories: light (≤10 g/day or lowest reported), heavy (>20 g/day or highest reported), and intermediate (values between these thresholds). These classifications were defined in accordance with international guidelines and widely adopted epidemiological standards [15]. The specific definitions for these categories, according to each included study, are provided in Supplementary Table 1. The risk of bias for all included retrospective and prospective observational studies was assessed using the Newcastle-Ottawa Scale (NOS), which evaluates selection, comparability, and outcome domains, assigning a maximum of 9 points. Studies were classified as having low, moderate, or high risk of bias accordingly [16].

### 2.2 Study objectives

Main objectives of the present analysis were to evaluate the incidence of BC in women with a history of alcohol consumption compared to those without history of alcohol consumption (cohort 1), and to assess the risk of BC recurrences and prognosis in patients with a history of alcohol consumption, compared to those without history of alcohol consumption (cohort 2).

Secondary objectives were to assess the association between the amount of alcohol consumption (i.e., light, intermediate, or heavy consumption) on BC incidence and on survival outcomes, to assess the association between alcohol consumption and the incidence of BC according to BC subtype (hormone receptor-positive vs. hormone receptor-negative diseases) and according to menopausal status at diagnosis (premenopausal vs. postmenopausal). All outcomes were evaluated using the longest follow-up data available in each study.

### 2.3 Statistical methods

Meta-analyses were performed by pooling RR, OR, and HR with their corresponding 95% CI, using random-effects models. Between-study heterogeneity was assessed using the Q statistic and quantified with the I^2^ statistic. Forest plots were generated to display individual study results and overall pooled estimates. Subgroup analyses were conducted for all endpoints based on the amount of alcohol intake (no, light, intermediate, or heavy consumption) and, for BC incidence, according to hormone receptor status and menopausal status. Publication bias was assessed using Egger's test where appropriate. A two-sided P-value of <0.05 was considered statistically significant. All statistical analyses were performed using Stata, software version 16.1 (StataCorp LLC, College Station, TX, USA).

## 3 Results

**Fig. 1:**
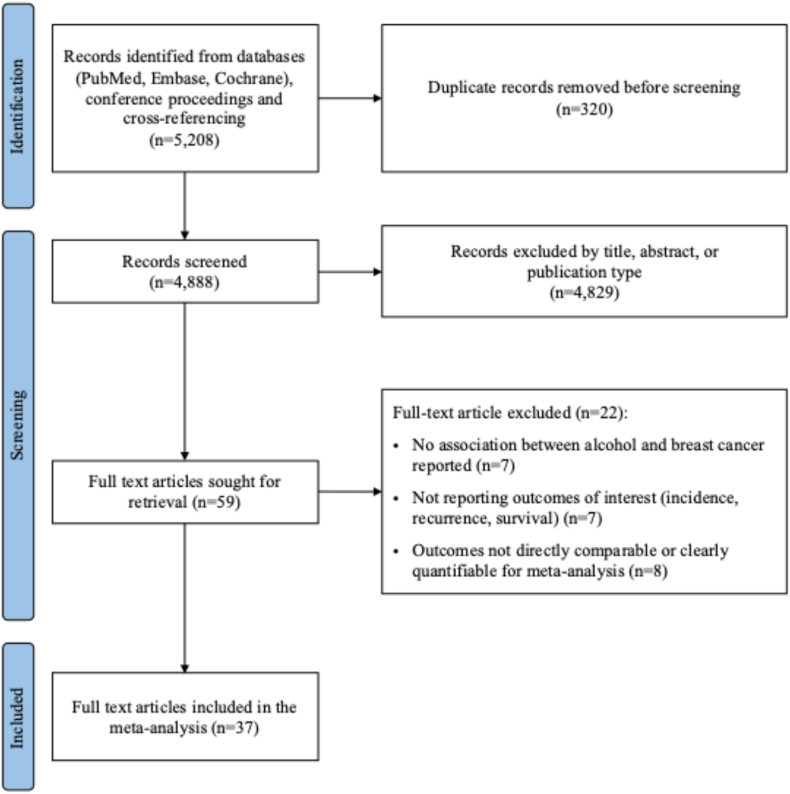
PRISMA flow diagram of study selection process.

Out of 5208 screened articles, after removing duplicates and applying the main inclusion and exclusion criteria, 59 full-text articles were assessed for eligibility. After excluding 22 full-text articles for not meeting the pre-specified criteria, 37 studies including 2,565,920 women were finally included in the present meta-analysis (Fig. 1). Of the 37 included studies, 30 were cohort studies and 7 were case-control studies. Among them, 17 studies[[17], [18], [19], [20], [21], [22], [23], [24], [25], [26], [27], [28], [29], [30], [31], [32], [33]] reported data on BC incidence in relation to alcohol consumption (cohort 1), while 20 studies [13,[34], [35], [36], [37], [38], [39], [40], [41], [42], [43], [44], [45], [46], [47], [48], [49], [50], [51], [52]] reported data on BC outcomes (cohort 2). The main characteristics of the included studies in cohort 1 and cohort 2 are summarized in Table 1 and in Table 2, respectively.

**Table 1:** Main characteristics of the studies assessing breast cancer incidence according to alcohol consumption included in the meta-analysis (Cohort 1).

First Author (Year of publication)	Years	Country	Study design	Type of alcoholic drink	Patients included in the study	Alcohol consumption among patients with BC		Timing of alcohol consumption assessment	Mean/median follow-up time	Outcomes evaluated	Risk of bias assessment
						Yes	No				
**Rohan T.E. et al. (2000)**	1980-1993	USA	Cohort	NR	56,837	1032	304	Before diagnosis	8/13 years	Incidence	High
**Feigelson H.S. et al. (2003)**	1992-1998	USA	Cohort	NR	66,561	705	598	Before diagnosis	5 years	Incidence	Moderate
**Mattisson I. et al. (2004)**	1991-2001	Sweden	Cohort	Beer	11,726	307	22	Before diagnosis	10 years	Incidence	Low
				Wine							
				Spirits							
**Suzuki R. et al. (2005)**	1987-1997	Sweden	Cohort	NR	51,847	970	314	Before diagnosis	8.3 years	Incidence	Low
**Zhang S.M. et al. (2007)**	1992-2004	USA	Cohort	NR	38,454	968	516	Before diagnosis	12 years	Incidence	Moderate
**Lew J.Q. et al. (2009)**	1995-2003	USA	Cohort	NR	184,418	3968	1493	Before and after diagnosis	7 years	Incidence	Moderate
**Allen N.E. et al. (2009)**	1996-2001	UK	Cohort	Wine (30%)	1,280,290	21,971	6409	Before diagnosis	7.2 years	Incidence	Moderate
				Beer/others (70%)							
**Li C.I. et al. (2010)**	1993-2005	USA	Cohort	Beer	87,724	2180	764	Before diagnosis	7/12 years	Incidence	Low
				Wine							
				Liquor							
**Chen W.Y.et al. (2011)**	1980-2008	USA	Cohort	NR	75,430	6021	1669	Before diagnosis	28 years	Incidence	Moderate
**Kawai M. et al. (2011)**	1990-2003	Japan	Cohort	NR	17,889	62	171	Before and after diagnosis	13 years	Incidence	Moderate
**Park S.Y. et al. (2014)**	1993-1996	USA	Cohort	Beer	85,935	1543	2343	Before diagnosis	12.4 years	Incidence	Moderate
				Wine							
				Liquor							
**Shin A. et al. (2015)**	1991-2009	Norway	Cohort	NR	45,233	1109	276	Before diagnosis	18 years	Incidence	Moderate
**Chhim A.S. et al. (2015)**	1994-1995	France	Cohort	Wine (81%)	3771	122	36	Before and after diagnosis	12.1 years	Incidence	High
				Beer and cider (9%)							
				Spirits (10%)							
**Romieu I. et al. (2015)**	1992-1998	France	Cohort	Wine	334,850	9950	1626	Before diagnosis	11 years	Incidence	Moderate
				Beer							
				Spirits							
				Liquors							
**Nitta J.et al. (2016)**	1990-2009	Japan	Cohort	NR	38,610	29	131	Before diagnosis	19 years	Incidence	High
**Kim H.J.et al. (2016)**	1991-2011	USA	Cohort	Wine	93,835	2060	806	Before diagnosis	20 years	Incidence	Moderate
				Beer							
				Liquor							
**Zeinomar N. et al. (2019)**	1992 - 2011	USA	Cohort	Beer	17,435	468	492	Before and after diagnosis	10.4 years	Incidence	Moderate
				Wine							
				Liquor							

Abbreviations: BC, breast cancer; NR, not reported.

**Table 2:** Main characteristics of the studies assessing breast cancer prognosis according to alcohol consumption included in the meta-analysis (Cohort 2).

First Author (ear of publication)	Years	Country	Study design	Types of alcoholic drink	Patients included in the study	Alcohol consumption among patients with BC outcomes		Timing of alcohol consumption	Mean/median follow-up time	Outcomes evaluated	Risk of bias assessment
						Yes	No				
**Jain M.G.et al. (2000)**	1980-1993	Canada	Cohort	Wine	48,942	174	49	Before diagnosis	10.3 years	Survival Outcomes	Moderate
				Beer							
				Liquor							
**Li C.I. et al. (2003)**	1992-2001	USA	Case-Control	NR	1256	46	31	Not specified	9/18 years	Recurrences	Low
**Reding K.W.et al. (2008)**	1983-2008	USA	Cohort	Wine	1284	1036	243	Before diagnosis	19 years	Survival Outcomes	Moderate
				Beer							
				Liquor							
**Knight J.A.et al. (2009)**	1985-2001	USA	Case-Control	Wine	2102	431	275	Before diagnosis	15 years	Recurrences	Moderate
				Cocktails Mixed							
				Spirits							
**Li C.I.et al. (2009)**	1990-2007	USA	Case-control	NR	854	142	712	Before diagnosis	NR	Recurrences	Low
**Hellman S.S.et al. (2010)**	1943-2003	Denmark	Cohort	NR	528	355	173	Before (60%) and after (40%) diagnosis	7.8 years	Survival Outcomes	High
**Flatt S.W.et al. (2010)**	1991-2000	USA	Case-control	Wine (47%)	3088	305	213	After diagnosis	7.3 years	Recurrences Survival Outcomes	Moderate
				Beer (23%)							
				Spirits (30%)							
**Kwan M.L. et al. (2010)**	1997-2000	USA	Cohort	Wine (88%)	1897	156	137	After diagnosis	10 years	Recurrences Survival Outcomes	Low
				Beer (35%)							
				Spirits (42%)							
**Harris H.R.et al. (2010)**	1987-2008	Sweden	Cohort	NR	3146	503	357	Before diagnosis (≈90%)	NR	Survival Outcomes	Low
**Vrieling A.et al. (2012)**	2001-2005	Germany	Cohort	Wine (97%)	2135	173	50	Before diagnosis	5 years	Survival Outcomes	Moderate
				Beer (66%)							
				Spirits/Liquor (57%)							
**Kwan M.L.et al. (2013)**	1993-2006	USA	Case-control	Wine	1487	863	624	After diagnosis	10.3 years	Recurrence Survival Outcomes	High
				Beer							
				Liquor							
**Holm M.et al. (2013)**	1993-1997	Denmark	Cohort	NR	1052	107	3	Before diagnosis	6.3 years	Recurrences Survival Outcomes	Moderate
**Newcomb P.A. et al. (2013)**	1998-2001	USA	Cohort	Beer	22,890	5929	1851	Before (≈80%) and after diagnosis (≈20%)	11.2 years	Survival Outcomes	Low
				Wine							
				Spirits							
**Weaver A.M.et al. (2013)**	1996-2001	USA	Case-control	Wine	1097	912	185	Before and after diagnosis	7.2 years	Survival Outcomes	High
				Beer							
				Liquors							
				Wine							
**Nechuta S.et al. (2015)**	1995-2006	USA/China	Cohort	NR	6596	634	529	After diagnosis	10.5/13.6 years	Recurrences Survival Outcomes	Low
**Din N.et al. (2016)**	1984-1988	USA	Case-control	Beer	939	518	421	Before diagnosis	11 years	Survival Outcomes	Moderate
				Wine							
				Other							
**Lowry S.J.et al. (2016)**	NR	USA	Cohort	NR	7835	883	135	Before and after diagnosis	7.9 years	Survival Outcomes	Low
**Ma H.et al. (2019)**	1994-1998	USA	Cohort	Beer	4523	586	489	Before diagnosis	8.6 years	Survival Outcomes	Low
				Wine							
				Liquor							
**Minami Y.et al. (2019)**	1997-2013	Japan	Cohort	Sake	1420	48	206	Before diagnosis	8.6 years	Survival Outcomes	Low
				Spirits							
				Beer							
				Wine							
**Zeinomar N. et al. (2023)**	2005-2019	USA	Cohort	Wine	1917	71	114	Before diagnosis	6.7 years	Survival Outcomes	Moderate
				Beer							
				Liquor							

Abbreviations: BC, breast cancer; NR, not reported.

### 3.1 Association between alcohol consumption and BC incidence (cohort 1)

The 17 studies evaluated in cohort 1 included overall 2,450,932 women, of whom 53,465 had a history of alcohol consumption and a subsequent diagnosis of BC, while 17,969 were diagnosed with BC in the context of absence of alcohol consumption [[17], [18], [19], [20], [21], [22], [23], [24], [25], [26], [27], [28], [29], [30], [31], [32], [33]]. The mean/median follow-up time of the studies ranged from 5 to 28 years.

**Fig. 2:**
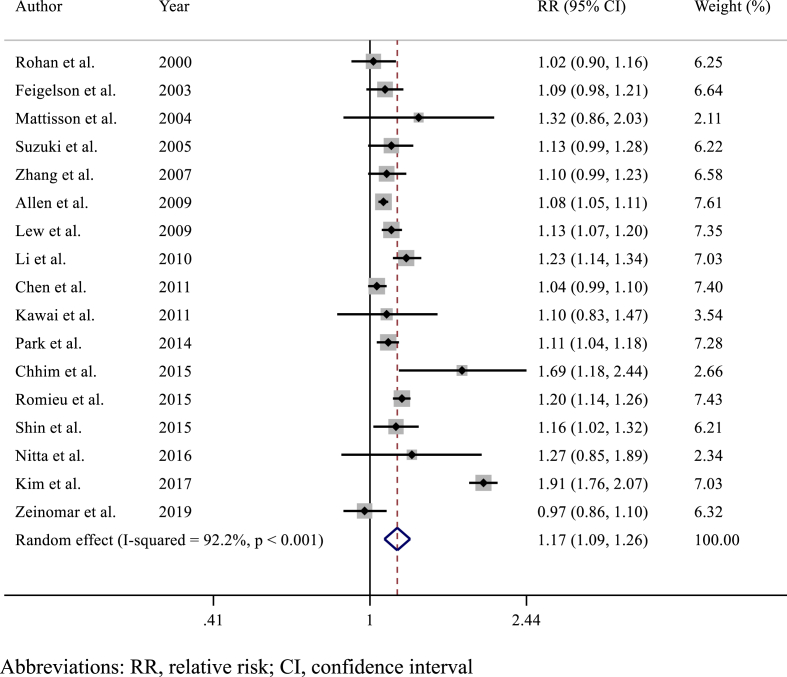
Forest plot showing the association between alcohol consumption and breast cancer incidence in the overall population Abbreviations: RR, relative risk; CI, confidence interval.

**Fig. 3:**
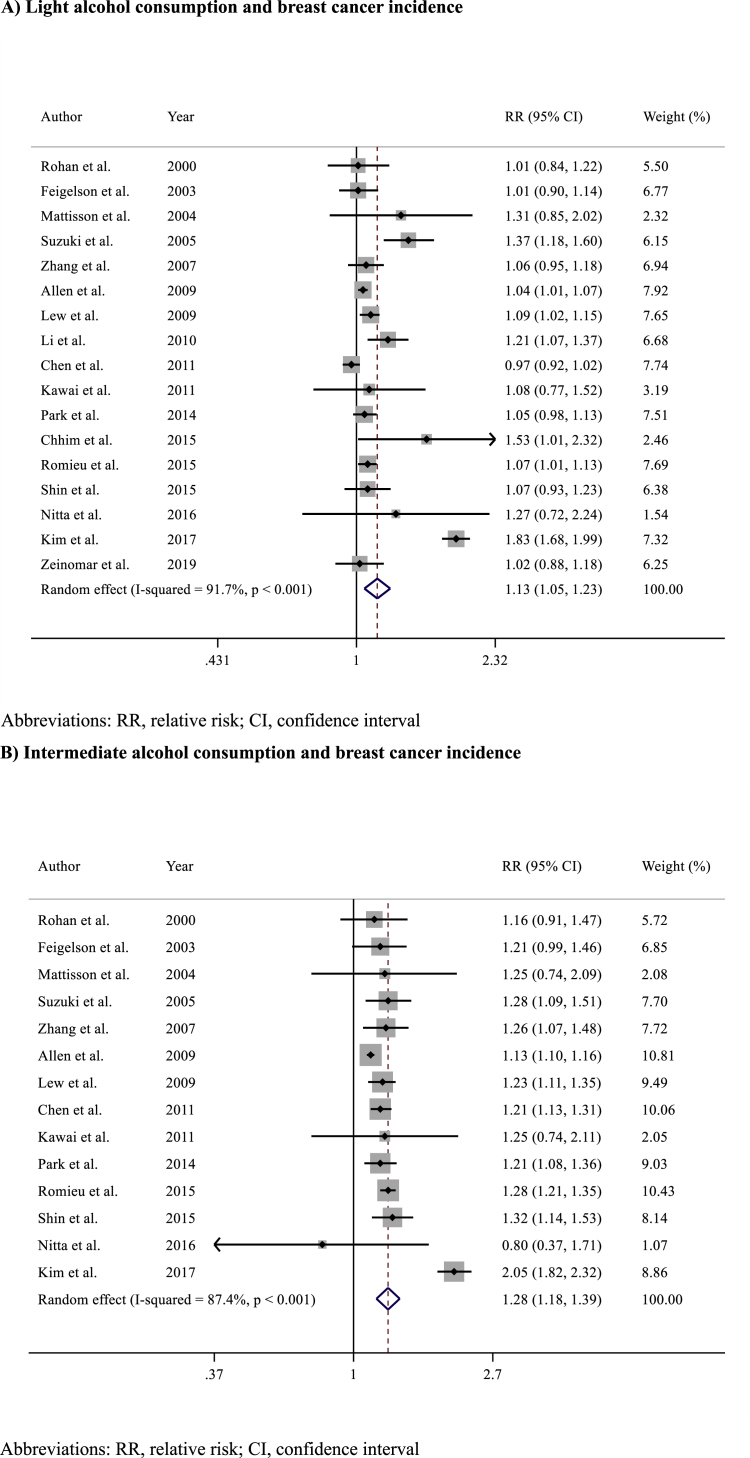

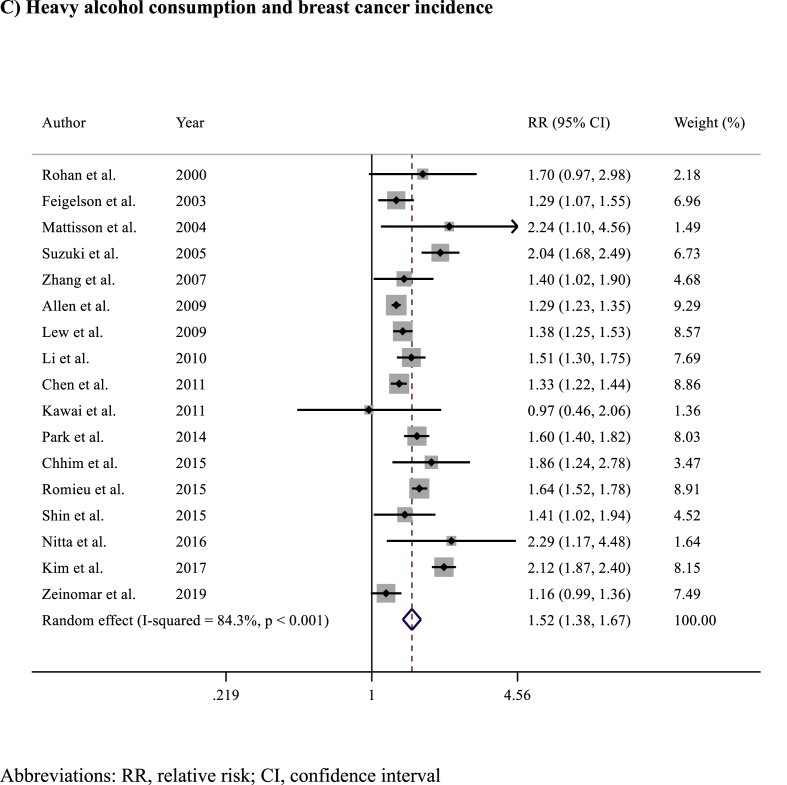
Forest plots showing the association between breast cancer incidence according to A) light B) intermediate or C) heavy alcohol consumption versus no alcohol consumption.

Alcohol consumption was associated with a statistically significant increased risk of BC incidence compared to no alcohol consumption (RR 1.17, 95% CI 1.09–1.26; p < 0.001) (Fig. 2). Significant heterogeneity was observed across the included studies (I^2^ = 92%, p < 0.001), but there was no evidence of publication bias (Egger's test p = 0.392) (Supplementary Table 2). The risk of BC incidence increased proportionally according to the levels of alcohol consumption: light alcohol consumption versus no consumption: RR 1.13 (95% CI 1.05–1.23; p = 0.002) (Fig. 3A)​, intermediate alcohol consumption versus no consumption: RR 1.28 (95% CI 1.18–1.39; p < 0.001)​ (Fig. 3B), and heavy alcohol consumption versus no consumption: RR 1.52 (95% CI 1.38–1.67; p < 0.001) (Fig. 3C).

Among the 9 studies (n = 921,326 women) reporting on the incidence of BC according to hormone receptor status, alcohol consumption appeared to be associated with an increased risk of hormone receptor-positive BC (RR 1.15, 95% CI 1.11–1.20; p < 0.001)​, while no significant association was found between alcohol consumption and the incidence of hormone receptor-negative diseases (RR 1.09, 95% CI 0.95–1.26; p = 0.212) (Supplementary Fig. 1A–B)​. Detailed sensitivity analyses are reported in Supplementary Tables 3–7.

### 3.2 Association between alcohol consumption and BC outcomes (cohort 2)

The 20 studies of cohort 2 included 114,988 patients with a previous history of BC diagnosis, of whom 13,872 had a history of alcohol consumption and a subsequent BC event (i.e., one event among recurrences and/or BCSS and/or OS) after BC diagnosis, while 6797 had a BC event after BC diagnosis without reporting history of alcohol consumption [13,[34], [35], [36], [37], [38], [39], [40], [41], [42], [43], [44], [45], [46], [47], [48], [49], [50], [51], [52]]. The mean/median follow-up time of the studies ranged from 5 years up to 19 years.

Among the 9 studies (n = 30,033 patients) reporting on BC recurrences, 17,403 patients had prior BC diagnosis and a history of alcohol consumption, while 12,630 patients had prior BC diagnosis without reporting any alcohol consumption. No significant association was found between alcohol consumption and BC recurrences (RR 1.02, 95% CI 0.93–1.11; p = 0.699; I^2^ = 42%, p = 0.087)​ (Supplementary Fig. 2). No evidence of publication bias was detected (Egger's test p = 0.860).

When analysing the different amounts of alcohol intake and BC recurrences, no significant associations were observed: light alcohol consumption versus no consumption RR 1.02 (95% CI 0.94–1.10; p = 0.635)​, intermediate alcohol consumption versus no consumption: RR 1.02 (95% CI 0.88–1.19; p = 0.770)​, heavy alcohol consumption versus no consumption: RR 1.12 (95% CI 0.99–1.26; p = 0.065). Forest plots and sensitivity analyses are reported in the Supplementary Figures 2, 3A-C, and Supplementary Tables 8–11.

Only 2 studies (n = 2,743) reported data regarding the risk of recurrence according to menopausal status, and no statistically significant differences were observed in the risk of recurrence between consumers and non-consumers among postmenopausal (RR 1.00, 95% CI 0.84–1.19) nor premenopausal (RR 0.86, 95% CI 0.73-1.01) patients (Supplementary Fig. 4A–B).

Among 13 studies (n = 61,099 patients) reporting on BCSS, no significant association was found between alcohol consumption and BCSS (HR 0.93, 95% CI 0.87–1.00; p = 0.050; I^2^ = 27%, p = 0.173)​ (Supplementary Fig. 5). No publication bias was detected (Egger's test p = 0.062). Subgroup analyses according to the amount of alcohol intake showed concordant results, without differences between light (HR 0.93; 95% CI 0.85–1.01; p = 0.083)​, intermediate (HR 0.91; 95% CI 0.81–1.03; p = 0.129)​, and heavy (HR 1.05; 95% CI 0.87–1.27; p = 0.621)​ alcohol consumption compared to no alcohol consumption (Supplementary Fig. 6 A-C and Supplementary Tables 12–15). No differences were observed in BCSS according to menopausal status (Supplementary Fig. 7A and B).

**Fig. 4:**
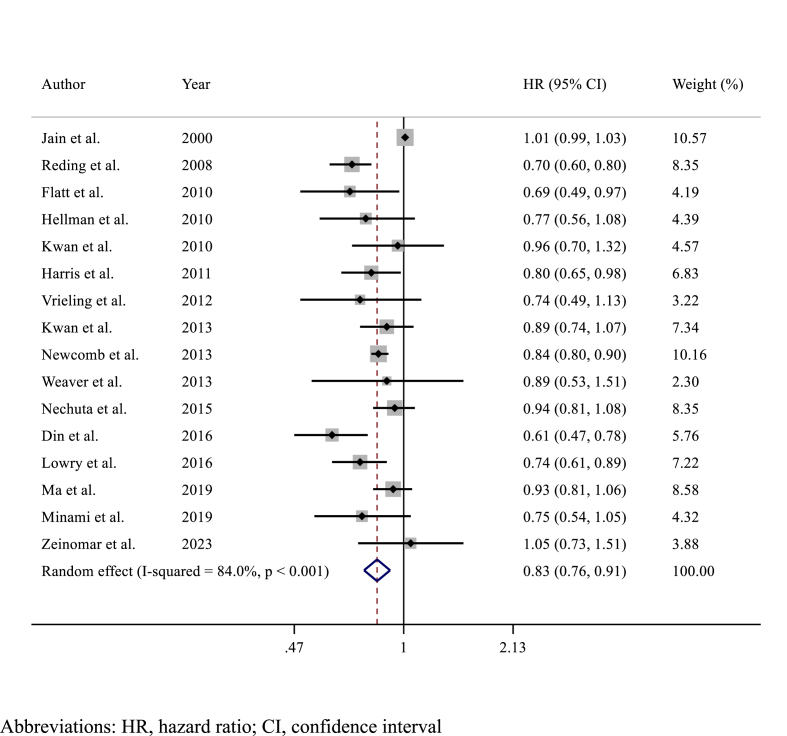
Forest plot showing the association between alcohol consumption and overall survival (OS) in breast cancer survivors Abbreviations: HR, hazard ratio; CI, confidence interval.

**Fig. 5:**
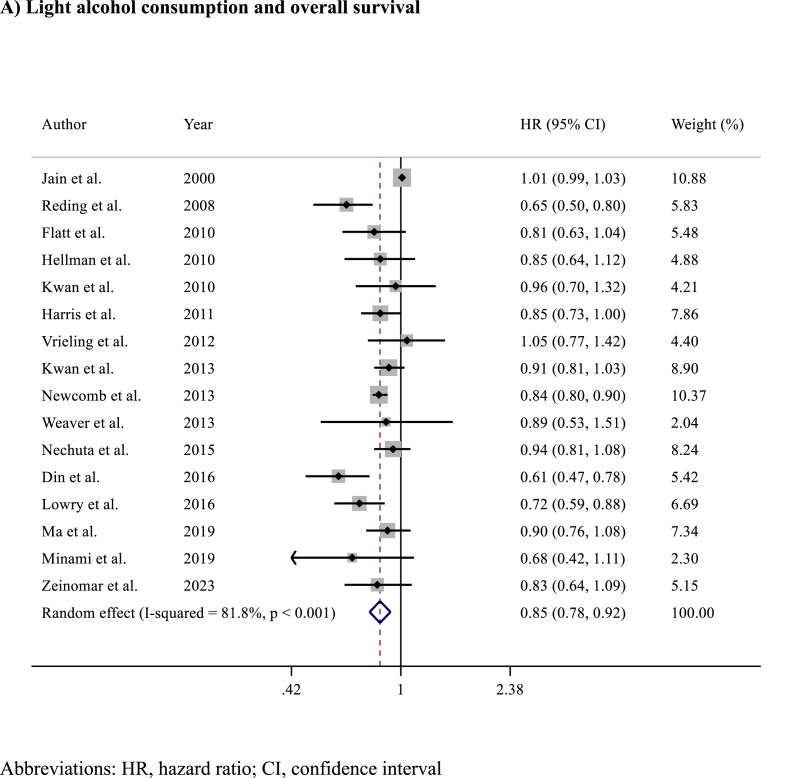

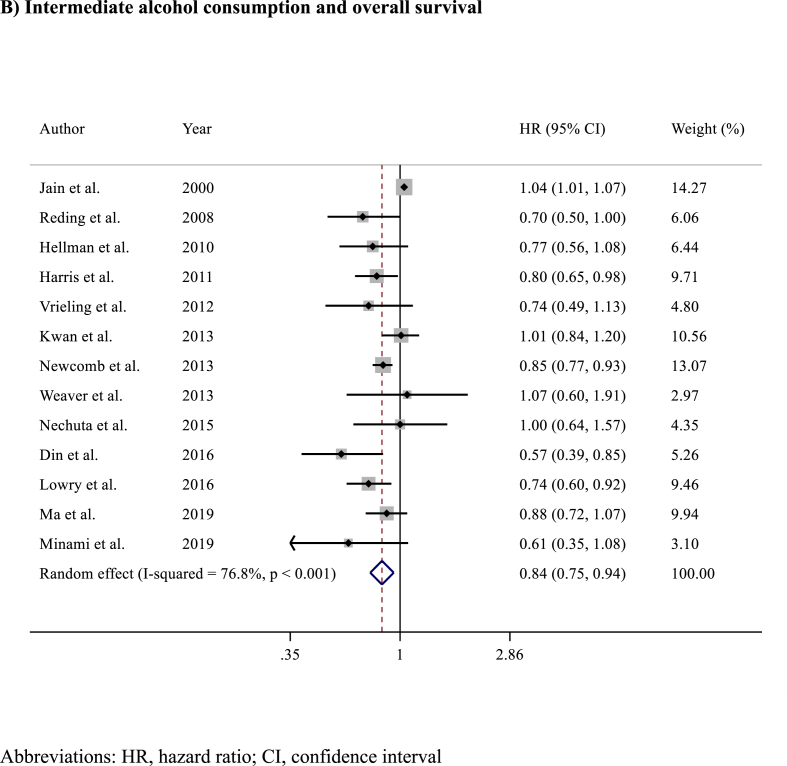

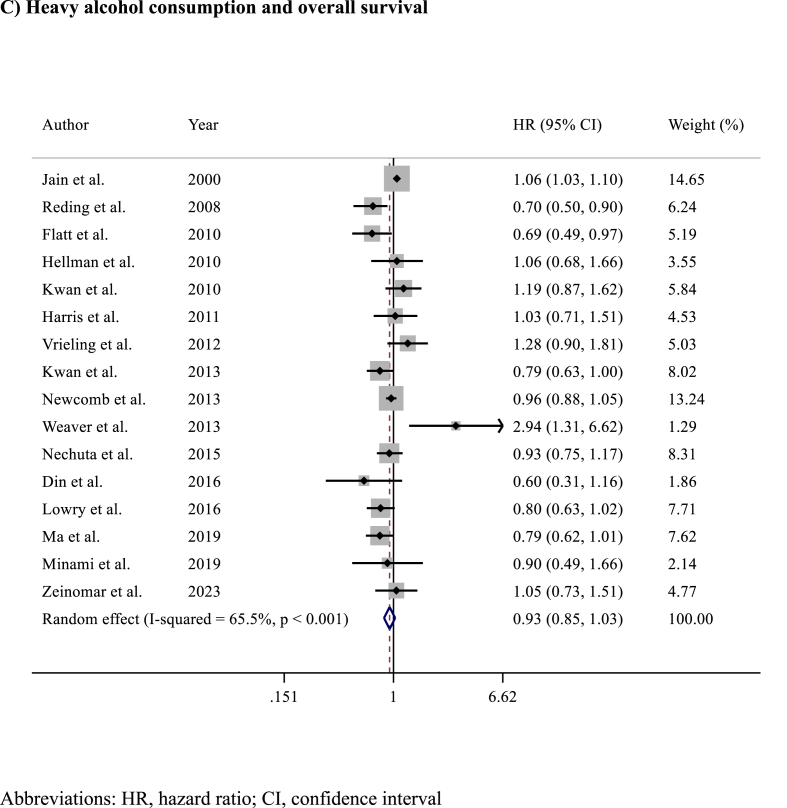
Forest plots showing the association between overall survival according to A) light B) intermediate or C) heavy alcohol consumption versus no alcohol consumption.

Among the 16 studies (n = 117,397 patients) reporting on prognosis in terms of OS, alcohol consumption was associated with slightly improved OS (HR 0.83, 95% CI 0.76–0.91; p < 0.001)​ (Fig. 4). Analysing OS results according to the amount of alcohol consumption, light (HR 0.85, 95% CI 0.78–0.92; p < 0.001)​ (Fig. 5A) and intermediate (HR 0.84, 95% CI 0.75–0.94; p = 0.002)​ (Fig. 5B) alcohol consumption were associated with improved OS compared to no consumption, while no differences in OS were observed between heavy alcohol consumption (HR 0.93, 95% CI 0.85–1.03; p = 0.20; I^2^ = 65%, p = 0.166) compared to no consumption​ (Fig. 5C). Detailed sensitivity analyses are presented in Supplementary Tables 16–19.

## 4 Discussion

The association between alcohol consumption and risk of BC has been explored in the last decades. However, inconsistencies persist, particularly regarding its impact on incidence according to hormone receptor status and on prognosis after BC diagnosis, as well as regarding the range in terms of intake amount, and prognosis after BC diagnosis. To further explore these inconsistencies, we conducted this meta-analysis that included 37 studies and over 2.5 million women.

Focusing on BC incidence, our meta-analysis confirmed previous findings by demonstrating that alcohol consumption is associated with an increased risk of developing BC [53]. Among 2,450,932 women included in the studies, those who had a previous history of alcohol consumption exhibited a 17% higher risk of BC incidence compared to non-consumers (RR 1.17; 95% CI 1.09–1.26). This finding may be explained by several biological mechanisms. First, alcohol is metabolized into acetaldehyde, a toxic and reactive compound capable of forming DNA adducts and inducing mutations that impair normal cellular repair mechanisms [54]. Second, alcohol consumption generates reactive oxygen species (ROS), leading to oxidative stress and further DNA damage [55]. Third, alcohol interferes with the absorption and metabolism of essential nutrients such as folate, an important factor for DNA synthesis and repair; folate deficiency has been independently associated with increased cancer risk [56]. Finally, alcohol intake elevates circulating estrogen levels [57,58], which is particularly relevant for the development of hormone receptor-positive BC [59]. Elevated estrogen promotes cellular proliferation and inhibits apoptosis, both processes facilitating carcinogenesis [60]. Collectively, these mechanisms underscore the biological plausibility of the association between alcohol intake and heightened BC risk [61]. Building on these biological mechanisms, emerging evidence suggests that alcohol consumption does not uniformly increase the risk of all BC subtypes. For example, a stronger association was observed particularly with hormone receptor-positive BC [62]. Several epidemiological studies demonstrated that alcohol-related BC risk is more pronounced among hormone receptor-positive subtypes, while the association with estrogen receptor-negative subtypes, such as triple-negative BC, appears to be weaker [63]. Our subgroup analysis supported this distinction, showing a significant association between alcohol consumption and increased risk of hormone receptor-positive BC (RR 1.15; 95% CI 1.11–1.20)​, while no significant association was found for hormone receptor-negative disease (RR 1.09; 95% CI 0.95–1.26). These findings reinforce the hypothesis that alcohol exerts its carcinogenic effects at least partially through hormonal pathways and emphasize the complexity of the impact of alcohol in BC development across different BC subtypes.

Given the biological bases, a dose-dependent carcinogenic effect of alcohol appears plausible. Our dose-response analysis confirmed this relationship, demonstrating a proportional increase in BC risk with higher levels of alcohol consumption: from a 13% relative risk increase in light alcohol consumers (RR 1.13), to a 28% increase among intermediate consumers (RR 1.28), and up to more than 50% higher risk among heavy consumers (RR 1.52). These findings are consistent with those reported in the literature. Bagnardi and colleagues described a progressive increase in BC risk, with risk ratios ranging from 1.04 (95% CI 1.01–1.07) among light consumers up to 1.61 (95% CI 1.33–1.94) among heavy consumers [53]. Similarly, Sun and colleagues reported a 10.5% rise in BC risk for each additional 10 g per day of alcohol consumption [64]. Despite the consistent association, it is important to recognize that the categorization of alcohol consumption into light, moderate (intermediate), and heavy consumption is not fully standardized and varies across epidemiological studies and clinical guidelines. Generally, light drinking is defined as consuming up to 10–12 g of pure alcohol per day (approximately equivalent to one standard drink), moderate intake ranges from 12 to 24 g per day, and heavy drinking exceeds 24–30 g daily. Organizations such as the World Health Organization (WHO), the World Cancer Research Fund (WCRF), and the American Cancer Society (ACS) recognize these thresholds, though slight variations exist depending on regional, cultural, and political contexts. For instance, The WCRF recommends limiting alcohol to one drink per day for women and two for men. In epidemiological research, alcohol intake is usually self-reported, which introduces heterogeneity in definitions and required some approximation in our meta-analysis. While this variability should be acknowledged when interpreting dose-response results, the overall consistency of findings across studies supports a biological gradient between alcohol consumption and BC risk.

Regarding the impact of personal history of alcohol consumption on BC prognosis, we evaluated three specific different endpoints: BC recurrences, BCSS, and OS. Overall, our meta-analysis found no significant association between alcohol consumption and the risk of recurrences (RR 1.02; p = 0.7). Similarly, no association has been observed between alcohol consumption on BCSS (HR 0.93; p = 0.5). These results were consistent across all subgroup analyses, including those focusing on heavy drinkers, and are aligned with prior results. Specifically, the meta-analysis by Gou and colleagues found no significant association between alcohol consumption on BC recurrences or BC related-mortality [65]. It is noteworthy that our study identified a potential protective effect of history of alcohol consumption (light and intermediate consumption) on OS among BC survivors. This observation, considering also the results in terms of BCSS, can be probably related and influenced by non-cancer-related deaths. Similar findings were reported by Barnett and colleagues in a cohort of 4560 women from the East Anglia Cancer Registry, reporting improved prognosis with increasing alcohol consumption, with a 2% reduction in the risk of death per unit of alcohol consumed per week [66]. Nomura et al. also observed similar improved OS results among women with pre-diagnosis alcohol consumption (RR 0.90; p = 0.02), while no differences were reported in terms of recurrences (RR 1.02; p = 0.88) or BCSS (RR 1.02; p = 0.7) [11]. These findings should not be interpreted as a recommendation to consume alcohol after BC diagnosis, since ethanol remains classified as a Group 1 carcinogen, and any apparent association with improved survival benefit should be interpreted with extreme caution, and the reasons for this apparent benefit are currently under investigation; however, different hypotheses have been proposed to explain this benefit in OS.

Several studies reported a potential reduction in overall mortality among patients consuming light amounts of alcohol. Bell and colleagues reported that in the UK, non-drinkers showed a significantly higher risk of cardiovascular events, including unstable angina, myocardial infarction, coronary death, heart failure, stroke, peripheral arterial disease, and abdominal aortic aneurysm compared to moderate drinkers. Conversely, heavy drinkers were also at increased risk for many cardiovascular outcomes, such as coronary death, heart failure, cardiac arrest, and various types of stroke [67]. The observed benefit has been mainly linked to certain alcoholic beverages, particularly for red wine [68,69]. However, the lack of a corresponding benefit in BCSS supports the hypothesis that the observed association with OS is primarily driven by non–breast cancer causes of death, particularly cardiovascular mortality, rather than by a direct protective effect on BC outcomes. These factors together seem to support the results observed in our meta-analysis. Moreover, residual confounding factors are likely to play a major role in the observed association between light and intermediate alcohol consumption and prognosis. Alcohol consumption is closely associated with socioeconomic status, dietary patterns, physical activity, and access to healthcare, all of which independently influence overall mortality and may not be fully accounted for in observational studies. Therefore, a causal relationship cannot be inferred. It should also be mentioned that we observed a higher incidence of hormone receptor-positive BC among alcohol consumers, and this could also partially explain a better OS for this specific disease subtype, although not demonstrated in terms of BCSS. This observation may further contribute to differences in OS without implying a direct effect of alcohol on BC biology. Overall, from a public health perspective, these results reinforce current recommendations advocating for alcohol reduction as a cancer prevention strategy and should not be interpreted as evidence supporting consumption among BC survivors [70].

There are several limitations in our analysis that warrant consideration. First, the method of alcohol exposure assessment varied considerably across the included studies, with most relying on self-reported questionnaires. Self-reported data are inherently subject to recall bias and social desirability bias, often resulting in underreporting of alcohol consumption, particularly in populations where drinking may carry social stigma [71]. This may have led to misclassification of exposure levels, potentially diluting true associations. Second, most included studies were observational, predominantly retrospective or prospective cohort studies, and are thus subject to a wide range of biases, including selection bias, information bias, and several other confounding factors. Third, there was a necessary approximation regarding the quantity of alcohol consumption, as studies used different thresholds to define “light,” “intermediate,” and “heavy” drinking categories. Despite efforts to harmonize these definitions across studies, residual heterogeneity may persist and could impact the precision of the dose-response results. Moreover, only few studies differentiated risk based on the type of alcoholic beverage consumed. This is particularly relevant considering that different beverages (e.g., red wine versus beers and spirits) have distinct compositions that could independently influence cancer risk and overall health outcomes [64]. Finally, few studies accounted for changes in alcohol consumption habits after a BC diagnosis. It is plausible that patients might modify their drinking behaviours after diagnosis, leading to potential exposure misclassification if only baseline or pre-diagnosis intake was considered [45]. Despite these limitations, this large meta-analysis including over 2.5 million women is, to our knowledge, the most up-to-date and comprehensive work attempting to establish the complex role between alcohol intake and risk of BC development or its prognosis after BC diagnosis.

In conclusion, our meta-analysis showed a strong dose-dependent association between alcohol consumption and BC incidence, particularly among patients diagnosed with hormone receptor-positive disease. In contrast, alcohol intake did not appear to significantly influence BC recurrence or BCSS and did not appear to worsen prognosis after a BC diagnosis. Any observed association with slightly improved survival should be interpreted cautiously, as it may reflect residual confounding and other biases related to study design rather than a true biological effect.

Overall, our findings reinforce the importance of alcohol reduction as a modifiable risk factor for BC prevention. Public health initiatives, such as the European Health Alliance on Alcohol, launched in 2025 by the WHO Regional Office for Europe [72], should continue to advocate for limiting alcohol consumption to mitigate cancer risk, while further research is needed to elucidate the complex relationship between alcohol intake and BC prognosis.

## CRediT authorship contribution statement

**Luca Arecco:** Writing – review & editing, Writing – original draft, Visualization, Validation, Supervision, Resources, Project administration, Methodology, Investigation, Data curation, Conceptualization. **Pedro M. Cacilhas:** Writing – review & editing, Writing – original draft, Visualization, Validation, Supervision, Resources, Project administration, Methodology, Investigation, Data curation, Conceptualization. **Camila Bobato Lara Gismondi:** Writing – review & editing, Writing – original draft, Visualization, Validation, Methodology, Investigation, Data curation. **Marco Bruzzone:** Writing – review & editing, Writing – original draft, Software, Formal analysis, Data curation. **Gabriella Gentile:** Writing – review & editing, Writing – original draft, Visualization, Data curation, Conceptualization. **Riccardo Gerosa:** Writing – review & editing, Writing – original draft, Visualization, Data curation. **Eva Blondeaux:** Writing – review & editing, Writing – original draft, Visualization, Software, Formal analysis, Data curation, Conceptualization. **Elisa Agostinetto:** Writing – review & editing, Writing – original draft, Visualization, Data curation, Conceptualization. **Chiara Dauccia:** Writing – review & editing, Writing – original draft, Visualization, Data curation, Conceptualization. **Soraia Lobo-Martins:** Writing – review & editing, Writing – original draft, Visualization, Data curation, Conceptualization. **Rafael Grochot:** Writing – review & editing, Writing – original draft, Visualization, Data curation, Conceptualization. **Kamal S. Saini:** Writing – review & editing, Writing – original draft, Visualization, Data curation, Conceptualization. **Hatem A. Azim:** Writing – review & editing, Writing – original draft, Visualization, Data curation, Conceptualization. **Marcio Debiasi:** Writing – review & editing, Writing – original draft, Visualization, Data curation, Conceptualization. **Alex De Caluwé:** Writing – review & editing, Writing – original draft, Visualization, Data curation, Conceptualization. **Laurence Buisseret:** Writing – review & editing, Writing – original draft, Visualization, Data curation, Conceptualization. **Lucia Del Mastro:** Writing – review & editing, Writing – original draft, Visualization, Data curation, Conceptualization. **Matteo Lambertini:** Writing – review & editing, Writing – original draft, Visualization, Supervision, Data curation, Conceptualization. **Evandro de Azambuja:** Writing – review & editing, Writing – original draft, Visualization, Validation, Supervision, Project administration, Methodology, Investigation, Data curation, Conceptualization.

## Data availability statement

All data supporting the findings of this study are derived from previously published articles and are publicly available. No individual participant data were collected or generated for this analysis. Summary data extracted from eligible studies are fully reported in the manuscript and its Supplementary Material.

## Funding

This research did not receive any specific grant from funding agencies in the public, commercial, or not-for-profit sectors.

## Declaration of competing interest

The authors declare the following financial interests/personal relationships which may be considered as potential competing interests: Luca Arecco reports travel grants from AstraZeneca and institutional research funding from Gilead Sciences. Eva Blondeaux reports speaker fees from Eli Lilly and research grants from Gilead Sciences.Elisa Agostinetto reports advisory role and/or honoraria from Eli Lilly, AstraZeneca, Bayer, Abscint, Gilead, Novartis; research grant to her institution from Gilead BeLux; meeting/travel grants from Novartis, Roche, Eli Lilly, Daiichi Sankyo, AstraZeneca, Abscint, Menarini, Gilead (all outside the submitted work). Soraia Lobo-Martins reports honoraria and/or advisory board fees from Roche, Novartis, Pfizer, Bristol Myers Squibb (BMS), AstraZeneca, MSD, and Gilead Sciences; conference support from Roche, Novartis, Daiichi Sankyo, AstraZeneca, BMS, Pierre Fabre, MSD, Lilly, Pfizer, Sanofi, Amgen, and Gilead Sciences; and fellowship participation in research funded institutionally by AstraZeneca, Novartis, and F. Hoffmann-La Roche Ltd at Institut Jules Bordet. Riccardo Gerosa Meeting/travel grants from Novartis and Daiichi Sankyo. Kamal S. Saini reports consulting fees from the European Commission and stock/ownership interests in Fortrea Inc. and Quantum Health Analytics (UK) Ltd, all outside the submitted work. Hatem A. Azim Jr. reports speaker fees from AstraZeneca and Roche and consultancy for PEP Therapy and Linkinvax. Marcio Debiasi reports speaker and/or consultancy fees from Roche, Lilly, Novartis, AstraZeneca, and Daiichi Sankyo; conference support from Roche, Gilead, Lilly, AstraZeneca, and Daiichi Sankyo; and an institutional research grant from MSD. Alex De Caluwé reports an institutional grant from AstraZeneca. Laurence Buisseret reports research funding to her institution from AstraZeneca, travel grants from Gilead, AstraZeneca, and Roche, and advisory roles for Domain Therapeutics, iTeos Therapeutics, AstraZeneca, and Roche. Lucia Del Mastro reports research grants from Eli Lilly, Novartis, Roche, Daiichi Sankyo, Seagen, AstraZeneca, Gilead, and Pierre Fabre; consulting fees from Eli Lilly, Gilead, and Daiichi Sankyo; speaker honoraria from multiple companies including Roche, Novartis, Pfizer, Eli Lilly, AstraZeneca, MSD, Seagen, Gilead, Pierre Fabre, Eisai, Exact Sciences, Ipsen, GSK, and Agendia-Stemline; travel grants from Roche, Pfizer, Eisai, Daiichi Sankyo, and AstraZeneca; and advisory roles for Novartis, Roche, Eli Lilly, Pfizer, Daiichi Sankyo, Exact Sciences, Gilead, Pierre Fabre, Eisai, AstraZeneca, Agendia, GSK, and Seagen. Matteo Lambertini reports advisory roles for Roche, Lilly, Novartis, AstraZeneca, Pfizer, Seagen, Gilead, MSD, Pierre Fabre, Exact Sciences, Menarini, and Nordic Pharma; speaker honoraria from Roche, Lilly, Novartis, Pfizer, Sandoz, Libbs, Daiichi Sankyo, Takeda, Knight, Ipsen, AstraZeneca, and Menarini; travel grants from Gilead, Roche, and Daiichi Sankyo; research funding to his institution from Gilead; and non-financial interests as a member of the National Council of the Italian Association of Medical Oncology. Evandro de Azambuja reports honoraria and/or advisory board participation from Roche/GNE, Novartis, Seagen, Zodiac, Libbs, Pierre Fabre, Lilly, AstraZeneca, MSD, and Gilead Sciences; travel grants from AstraZeneca and Gilead; institutional research grants from Roche/GNE, AstraZeneca, GSK/Novartis, Gilead Sciences, and Seagen/Pfizer; and non-financial roles as ESMO Director of Membership (2023–2025) and BSMO President (2023–2026). All remaining authors declare no competing interests. If there are other authors, they declare that they have no known competing financial interests or personal relationships that could have appeared to influence the work reported in this paper.
