# Supplementary material for: Association between alcohol consumption and breast cancer incidence and prognosis: A systematic review and meta-analysis
Source: Breast. 2026 Feb 5;86:104719. doi: 10.1016/j.breast.2026.104719 (PMC12925132; doi:10.1016/j.breast.2026.104719)
Supplement: Multimedia component 1 [file mmc1.docx]

**Supplementary Material**

[Supplementary Figure 1. Forest plot showing the association between alcohol consumption and the risk of A) Hormone receptor-positive and B) Hormone receptor-negative breast cancer 3](#_Toc205813843)

[A) Hormone receptor-positive breast cancer 3](#_Toc205813844)

[B) Hormone receptor-negative breast cancer 4](#_Toc205813845)

[Supplementary Figure 2. Forest plot showing the association between alcohol consumption and breast cancer recurrence 5](#_Toc205813846)

[Supplementary Figure 3. Forest plot showing the association between breast cancer recurrence according to A) Light B) Intermediate or C) Heavy alcohol consumption versus no alcohol consumption 6](#_Toc205813847)

[A) Light alcohol consumption and breast cancer recurrence 6](#_Toc205813848)

[B) Intermediate alcohol consumption and breast cancer recurrence 7](#_Toc205813849)

[C) Heavy alcohol consumption and breast cancer recurrence 8](#_Toc205813850)

[Supplementary Figure 4. Forest plot showing the association between breast cancer recurrence and alcohol consumption according to menopausal status A) Premenopausal B) Postmenopausal 9](#_Toc205813851)

[A) Premenopausal 9](#_Toc205813852)

[B) Postmenopausal 10](#_Toc205813853)

[Supplementary Figure 5. Forest plot showing the association between alcohol consumption and breast cancer specific survival 11](#_Toc205813854)

[Supplementary Figure 6. Forest plot showing the association between breast cancer specific survival according to A) light B) intermediate or C) heavy alcohol consumption versus no alcohol consumption 12](#_Toc205813855)

[A) Light alcohol consumption and breast cancer specific survival 12](#_Toc205813856)

[B) Intermediate alcohol consumption and breast cancer specific survival 13](#_Toc205813857)

[C) Heavy alcohol consumption and breast cancer specific survival 14](#_Toc205813858)

[Supplementary Figure 7. Forest plot showing the association between breast cancer specific survival and alcohol consumption according to menopausal status A) Premenopausal B) Postmenopausal 15](#_Toc205813859)

[A) Premenopausal 15](#_Toc205813860)

[B) Postmenopausal 16](#_Toc205813861)

[Supplementary Table 1. Specific definitions for Light, Intermediate, and Heavy alcohol consumption categories according to each included study 17](#_Toc205813862)

[Supplementary Table 2. Sensitivity analysis for the association between alcohol consumption and breast cancer incidence 19](#_Toc205813863)

[Supplementary Table 3. Sensitivity analysis for the association between light alcohol consumption and breast cancer incidence 20](#_Toc205813864)

[Supplementary Table 4. Sensitivity analysis for the association between intermediate alcohol consumption and breast cancer incidence 21](#_Toc205813865)

[Supplementary Table 5. Sensitivity analysis for the association between heavy alcohol consumption and breast cancer incidence 22](#_Toc205813866)

[Supplementary Table 6. Sensitivity analysis for the association between alcohol consumption and hormone receptor-positive breast cancer incidence 23](#_Toc205813867)

[Supplementary Table 7. Sensitivity analysis for the association between alcohol consumption and hormone receptor-negative breast cancer incidence 24](#_Toc205813868)

[Supplementary Table 8. Sensitivity analysis for the association between alcohol consumption and breast cancer recurrences 25](#_Toc205813869)

[Supplementary Table 9. Sensitivity analysis for the association between light alcohol consumption and breast cancer recurrences 26](#_Toc205813870)

[Supplementary Table 10. Sensitivity analysis for the association between intermediate alcohol consumption and breast cancer recurrences 27](#_Toc205813871)

[Supplementary Table 11. Sensitivity analysis for the association between heavy alcohol consumption and breast cancer recurrences 28](#_Toc205813872)

[Supplementary Table 12. Sensitivity analysis for the association between alcohol consumption and breast cancer-specific survival 29](#_Toc205813873)

[Supplementary Table 13. Sensitivity analysis for the association between light alcohol consumption and breast cancer-specific survival 30](#_Toc205813874)

[Supplementary Table 14. Sensitivity analysis for the association between intermediate alcohol consumption and breast cancer-specific survival 31](#_Toc205813875)

[Supplementary Table 15. Sensitivity analysis for the association between heavy alcohol consumption and breast cancer-specific survival 32](#_Toc205813876)

[Supplementary Table 16. Sensitivity analysis for the association between alcohol consumption and overall survival 33](#_Toc205813877)

[Supplementary Table 17. Sensitivity analysis for the association between light alcohol consumption and overall survival 34](#_Toc205813878)

[Supplementary Table 18. Sensitivity analysis for the association between intermediate alcohol consumption and overall survival 35](#_Toc205813879)

[Supplementary Table 19. Sensitivity analysis for the association between heavy alcohol consumption and overall survival 36](#_Toc205813880)

# **Supplementary Figure 1. Forest plot showing the association between alcohol consumption and the risk of A) Hormone receptor-positive and B) Hormone receptor-negative breast cancer**

## **A) Hormone receptor-positive breast cancer**

Abbreviations: RR, relative risk; CI, confidence interval

## **B) Hormone receptor-negative breast cancer**

Abbreviations: RR, relative risk; CI, confidence interval

# **Supplementary Figure 2. Forest plot showing the association between alcohol consumption and breast cancer recurrence**

Abbreviations: RR, relative risk; CI, confidence interval

# **Supplementary Figure 3. Forest plot showing the association between breast cancer recurrence according to A) Light B) Intermediate or C) Heavy alcohol consumption versus no alcohol consumption**

## **A) Light alcohol consumption and breast cancer recurrence**

Abbreviations: HR, hazard ratio; CI, confidence interval

## **B) Intermediate alcohol consumption and breast cancer recurrence**

Abbreviations: HR, hazard ratio; CI, confidence interval

## **C) Heavy alcohol consumption and breast cancer recurrence**

Abbreviations: HR, hazard ratio; CI, confidence interval

# **Supplementary Figure 4. Forest plot showing the association between breast cancer recurrence and alcohol consumption according to menopausal status A) Premenopausal B) Postmenopausal**

## **A) Premenopausal**

Abbreviations: RR, relative risk; CI, confidence interval

## **B) Postmenopausal**

Abbreviations: RR, relative risk; CI, confidence interval

# **Supplementary Figure 5. Forest plot showing the association between alcohol consumption and breast cancer specific survival**

Abbreviations: HR, hazard ratio; CI, confidence interval

# **Supplementary Figure 6. Forest plot showing the association between breast cancer specific survival according to A) light B) intermediate or C) heavy alcohol consumption versus no alcohol consumption**

## **A) Light alcohol consumption and breast cancer specific survival**

Abbreviations: HR, hazard ratio; CI, confidence interval

## **B) Intermediate alcohol consumption and breast cancer specific survival**

Abbreviations: HR, hazard ratio; CI, confidence interval

## **C) Heavy alcohol consumption and breast cancer specific survival**

Abbreviations: HR, hazard ratio; CI, confidence interval

# **Supplementary Figure 7. Forest plot showing the association between breast cancer specific survival and alcohol consumption according to menopausal status A) Premenopausal B) Postmenopausal**

## **A) Premenopausal**

Abbreviations: HR, hazard ratio; CI, confidence interval

## **B) Postmenopausal**

Abbreviations: HR, hazard ratio; CI, confidence interval

# **Supplementary Table 1. Specific definitions for Light, Intermediate, and Heavy alcohol consumption categories according to each included study**

|  | **Baseline studies information and characteristics** | | | **Alcohol intake categories*** | | |
| --- | --- | --- | --- | --- | --- | --- |
|  | **First author** | **Year** | **Country** | **Light**  **(g/day)** | **Intermediate**  **(g/day)** | **Heavy**  **(g/day)** |
| **COHORT 1** | | | | | | |
| 1 | Rohan T.E. | 2000 | Canada | <10 | 10-20 | >20 |
| 2 | Feigelson H.S. | 2003 | USA | <10 | 10-15 | >15 |
| 3 | Mattisson I. | 2004 | Sweden | <15 | 15-30 | >30 |
| 4 | Suzuki R. | 2005 | Sweden | <3.5 | 3.5-10 | >10 |
| 5 | Zhang S.M. | 2007 | USA | <10 | 10-30 | >30 |
| 6 | Lew | 2009 | USA | <10 | 10-20 | >20 |
| 7 | Allen N.E. | 2009 | UK | <10 | 10-20 | >20 |
| 8 | Li C. | 2010 | USA | <5 | 5-20 | >20 |
| 9 | Chen W.Y. | 2011 | USA | <10 | 10-20 | >20 |
| 10 | Kawai M. | 2011 | Japan | < 5 | 5-15 | >15 |
| 11 | Park S.Y. | 2014 | USA | <10 | 10-30 | >30 |
| 12 | Shin A. | 2015 | Norway | <5 | 5-15 | >15 |
| 13 | Chimm A.S. | 2015 | France | Lower tertile | NR | Higher tertile |
| 14 | Romieu I. | 2015 | France | <5 | 5-30 | >30 |
| 15 | Nitta J. | 2016 | Japan | <5 | 5-15 | >15 |
| 16 | Kim H.J. | 2017 | USA | <5 | 5-10 | >10 |
| 17 | Zeinomar N. | 2019 | USA | <10 | NR | >10 |
| **COHORT 2** | | | | | | |
| 1 | Jain M.G. | 2000 | Canada | <10 | 10-20 | >20 |
| 2 | Li C.I. | 2003 | USA | <10 | NR | >10 |
| 3 | Reding K.W. | 2008 | USA | <4 | 4-10 | >10 |
| 4 | Knight J.A. | 2008 | USA | <10 | NR | >10 |
| 5 | Li C. | 2009 | USA | <10 | NR | >10 |
| 6 | Hellman S.S. | 2010 | Denmark | <8.5 | 8.5-20 | >20 |
| 7 | Flatt S.W. | 2010 | USA | <10 | NR | >10 |
| 8 | Kwan M.L. | 2010 | USA | <6 | NR | >6 |
| 9 | Harris H.R. | 2012 | Sweden | <3.5 | 3.5-10 | >10 |
| 10 | Vrieling A. | 2012 | Germany | <6 | 6-12 | >12 |
| 11 | Kwan M.L. | 2013 | USA | <6 | 6-12 | >12 |
| 12 | Holm M. | 2013 | Denmark | <10 | 10-20 | >20 |
| 13 | Newcomb P.A. | 2013 | USA | <3 | 3-13 | >13 |
| 14 | Weaver A.M. | 2013 | USA | <10 | 20-30 | >30 |
| 15 | Nechuta S. | 2015 | USA/China | <6 | 6-12 | >12 |
| 16 | Din N. | 2016 | USA | <5.5 | 5.5-14 | >14 |
| 17 | Lowry S.J. | 2018 | USA | <1.5 | 1.5 – 8.5 | >8.5 |
| 18 | Ma H. | 2019 | USA | <4.5 | 4.5-10 | >10 |
| 19 | Minami | 2019 | Japan | <5 | NR | >5 |
| 20 | Zeinomar N. | 2023 | USA | <5 | NR | >5 |

*To harmonize the categorization of alcohol intake across studies with varying definitions and reporting formats, we adopted a standardized classification based on commonly accepted epidemiological thresholds and, when needed, approximated intake levels using study-specific percentiles. Specifically, light alcohol consumption was defined as consumption of up to 10 grams of pure alcohol per day, or the lowest tertile of intake when intake was reported in quantiles. Intermediate consumption was defined as between 10 and 20 grams per day, while heavy consumption was defined as greater than 20 grams per day, or the highest tertile of intake, depending on the data available. When alcohol consumption was reported in categorical or qualitative terms (i.e., “low,” “moderate,” or “high”), categories were matched to these gram-based thresholds as closely as possible.

# **Supplementary Table 2. Sensitivity analysis for the association between alcohol consumption and breast cancer incidence**

| **Study excluded** | **Random effect** | | | **I-squared (%)** | **I-sq. P-value** |
| --- | --- | --- | --- | --- | --- |
|  | **RR** | **95% CI** | **P-value** |  |  |
| Rohan et al. 2000 | 1.18 | 1.09-1.27 | <0.001 | 92.6 | <0.001 |
| Feigelson et al. 2003 | 1.18 | 1.09-1.27 | <0.001 | 92.7 | <0.001 |
| Mattisson et al. 2004 | 1.17 | 1.08-1.26 | <0.001 | 92.7 | <0.001 |
| Suzuki et al. 2005 | 1.17 | 1.09-1.27 | <0.001 | 92.7 | <0.001 |
| Zhang et al. 2007 | 1.17 | 1.09-1.27 | <0.001 | 92.7 | <0.001 |
| Allen et al. 2009 | 1.18 | 1.08-1.29 | <0.001 | 92.0 | <0.001 |
| Lew et al. 2009 | 1.17 | 1.08-1.27 | <0.001 | 92.7 | <0.001 |
| Li et al. 2010 | 1.17 | 1.08-1.26 | <0.001 | 92.5 | <0.001 |
| Chen et al. 2011 | 1.18 | 1.09-1.28 | <0.001 | 92.3 | <0.001 |
| Kawai et al. 2011 | 1.17 | 1.09-1.26 | <0.001 | 92.7 | <0.001 |
| Park et al. 2014 | 1.17 | 1.08-1.27 | <0.001 | 92.7 | <0.001 |
| Chhim et al. 2015 | 1.16 | 1.08-1.25 | <0.001 | 92.5 | <0.001 |
| Romieu et al. 2015 | 1.17 | 1.08-1.27 | <0.001 | 92.5 | <0.001 |
| Shin et al. 2015 | 1.17 | 1.08-1.26 | <0.001 | 92.7 | <0.001 |
| Nitta et al. 2016 | 1.17 | 1.08-1.26 | <0.001 | 92.7 | <0.001 |
| Kim et al. 2017 | 1.12 | 1.08-1.16 | <0.001 | 61.0 | 0.001 |
| Zeinomar et al. 2019 | 1.18 | 1.10-1.28 | <0.001 | 92.5 | <0.001 |

Abbreviations: RR, relative risk; CI, confidence interval

# **Supplementary Table 3. Sensitivity analysis for the association between light alcohol consumption and breast cancer incidence**

| **Study excluded** | **Random effect** | | | **I-squared (%)** | **I-sq. P-value** |
| --- | --- | --- | --- | --- | --- |
|  | **RR** | **95% CI** | **P-value** |  |  |
| Rohan et al. 2000 | 1.14 | 1.05-1.24 | 0.001 | 92.2 | <0.001 |
| Feigelson et al. 2003 | 1.14 | 1.05-1.24 | 0.001 | 92.1 | <0.001 |
| Mattisson et al. 2004 | 1.13 | 1.04-1.22 | 0.002 | 92.1 | <0.001 |
| Suzuki et al. 2005 | 1.12 | 1.03-1.21 | 0.005 | 91.8 | <0.001 |
| Zhang et al. 2007 | 1.14 | 1.05-1.24 | 0.002 | 92.2 | <0.001 |
| Allen et al. 2009 | 1.15 | 1.04-1.26 | 0.005 | 91.8 | <0.001 |
| Lew et al. 2009 | 1.14 | 1.05-1.24 | 0.003 | 92.2 | <0.001 |
| Li et al. 2010 | 1.13 | 1.04-1.23 | 0.004 | 92.0 | <0.001 |
| Chen et al. 2011 | 1.15 | 1.06-1.25 | 0.001 | 91.4 | <0.001 |
| Kawai et al. 2011 | 1.14 | 1.05-1.23 | 0.002 | 92.2 | <0.001 |
| Park et al. 2014 | 1.14 | 1.05-1.24 | 0.003 | 92.2 | <0.001 |
| Chhim et al. 2015 | 1.13 | 1.04-1.22 | 0.003 | 92.1 | <0.001 |
| Romieu et al. 2015 | 1.14 | 1.05-1.25 | 0.003 | 92.2 | <0.001 |
| Shin et al. 2015 | 1.14 | 1.05-1.24 | 0.002 | 92.2 | <0.001 |
| Nitta et al. 2016 | 1.13 | 1.05-1.23 | 0.002 | 92.2 | <0.001 |
| Kim et al. 2017 | 1.07 | 1.03-1.11 | 0.001 | 55.4 | 0.004 |
| Zeinomar et al. 2019 | 1.14 | 1.05-1.24 | 0.001 | 92.2 | <0.001 |

Abbreviations: RR, relative risk; CI, confidence interval

# **Supplementary Table 4. Sensitivity analysis for the association between intermediate alcohol consumption and breast cancer incidence**

| **Study excluded** | **Random effect** | | | **I-squared (%)** | **I-sq. P-value** |
| --- | --- | --- | --- | --- | --- |
|  | **RR** | **95% CI** | **P-value** |  |  |
| Rohan et al. 2000 | 1.29 | 1.18-1.40 | <0.001 | 88.4 | <0.001 |
| Feigelson et al. 2003 | 1.29 | 1.18-1.40 | <0.001 | 88.4 | <0.001 |
| Mattisson et al. 2004 | 1.28 | 1.18-1.39 | <0.001 | 88.4 | <0.001 |
| Suzuki et al. 2005 | 1.28 | 1.17-1.40 | <0.001 | 88.3 | <0.001 |
| Zhang et al. 2007 | 1.28 | 1.17-1.40 | <0.001 | 88.4 | <0.001 |
| Allen et al. 2009 | 1.30 | 1.19-1.42 | <0.001 | 81.2 | <0.001 |
| Lew et al. 2009 | 1.28 | 1.17-1.41 | <0.001 | 88.4 | <0.001 |
| Chen et al. 2011 | 1.29 | 1.17-1.41 | <0.001 | 88.4 | <0.001 |
| Kawai et al. 2011 | 1.28 | 1.18-1.39 | <0.001 | 88.4 | <0.001 |
| Park et al. 2014 | 1.29 | 1.18-1.41 | <0.001 | 88.4 | <0.001 |
| Romieu et al. 2015 | 1.28 | 1.16-1.41 | <0.001 | 87.5 | <0.001 |
| Shin et al. 2015 | 1.28 | 1.17-1.39 | <0.001 | 88.2 | <0.001 |
| Nitta et al. 2016 | 1.29 | 1.18-1.40 | <0.001 | 88.3 | <0.001 |
| Kim et al. 2017 | 1.22 | 1.16-1.28 | <0.001 | 51.5 | 0.016 |

Abbreviations: RR, relative risk; CI, confidence interval

# **Supplementary Table 5. Sensitivity analysis for the association between heavy alcohol consumption and breast cancer incidence**

| **Study excluded** | **Random effect** | | | **I-squared (%)** | **I-sq. P-value** |
| --- | --- | --- | --- | --- | --- |
|  | **RR** | **95% CI** | **P-value** |  |  |
| Rohan et al. 2000 | 1.52 | 1.38-1.67 | <0.001 | 85.3 | <0.001 |
| Feigelson et al. 2003 | 1.54 | 1.39-1.70 | <0.001 | 85.2 | <0.001 |
| Mattisson et al. 2004 | 1.51 | 1.37-1.66 | <0.001 | 85.1 | <0.001 |
| Suzuki et al. 2005 | 1.49 | 1.35-1.63 | <0.001 | 83.2 | <0.001 |
| Zhang et al. 2007 | 1.53 | 1.38-1.68 | <0.001 | 85.3 | <0.001 |
| Allen et al. 2009 | 1.54 | 1.40-1.71 | <0.001 | 79.5 | <0.001 |
| Lew et al. 2009 | 1.53 | 1.38-1.70 | <0.001 | 85.3 | <0.001 |
| Li et al. 2010 | 1.52 | 1.37-1.68 | <0.001 | 85.2 | <0.001 |
| Chen et al. 2011 | 1.54 | 1.39-1.71 | <0.001 | 84.9 | <0.001 |
| Kawai et al. 2011 | 1.53 | 1.39-1.68 | <0.001 | 85.2 | <0.001 |
| Park et al. 2014 | 1.51 | 1.37-1.67 | <0.001 | 84.9 | <0.001 |
| Chhim et al. 2015 | 1.51 | 1.37-1.66 | <0.001 | 85.1 | <0.001 |
| Romieu et al. 2015 | 1.51 | 1.36-1.67 | <0.001 | 83.0 | <0.001 |
| Shin et al. 2015 | 1.52 | 1.38-1.68 | <0.001 | 85.3 | <0.001 |
| Nitta et al. 2016 | 1.51 | 1.37-1.66 | <0.001 | 85.0 | <0.001 |
| Kim et al. 2017 | 1.46 | 1.35-1.59 | <0.001 | 75.4 | <0.001 |
| Zeinomar et al. 2019 | 1.55 | 1.41-1.71 | <0.001 | 84.3 | <0.001 |

Abbreviations: RR, relative risk; CI, confidence interval

# **Supplementary Table 6. Sensitivity analysis for the association between alcohol consumption and hormone receptor-positive breast cancer incidence**

| **Study excluded** | **Random effect** | | | **I-squared (%)** | **I-sq. P-value** |
| --- | --- | --- | --- | --- | --- |
|  | **RR** | **95% CI** | **P-value** |  |  |
| Suzuki et al. 2005 | 1.15 | 1.10-1.21 | <0.001 | 34.7 | 0.151 |
| Zhang et al. 2007 | 1.16 | 1.11-1.21 | <0.001 | 29.6 | 0.192 |
| Lew et al. 2009 | 1.14 | 1.10-1.19 | <0.001 | 26.4 | 0.218 |
| Li et al. 2010 | 1.13 | 1.09-1.17 | <0.001 | 0.0 | 0.819 |
| Chen et al. 2011 | 1.17 | 1.12-1.22 | <0.001 | 17.4 | 0.293 |
| Park et al. 2014 | 1.16 | 1.11-1.21 | <0.001 | 32.0 | 0.173 |
| Romieu et al. 2015 | 1.16 | 1.10-1.21 | <0.001 | 34.3 | 0.154 |
| Shin et al. 2015 | 1.15 | 1.10-1.20 | <0.001 | 33.7 | 0.159 |
| Zeinomar et al. 2019 | 1.16 | 1.11-1.21 | <0.001 | 32.0 | 0.173 |

Abbreviations: RR, relative risk; CI, confidence interval

# **Supplementary Table 7. Sensitivity analysis for the association between alcohol consumption and hormone receptor-negative breast cancer incidence**

| **Study excluded** | **Random effect** | | | **I-squared (%)** | **I-sq. P-value** |
| --- | --- | --- | --- | --- | --- |
|  | **RR** | **95% CI** | **P-value** |  |  |
| Suzuki et al. 2005 | 1.09 | 0.94-1.27 | 0.268 | 72.7 | 0.001 |
| Zhang et al. 2007 | 1.08 | 0.93-1.27 | 0.301 | 72.7 | 0.001 |
| Lew et al. 2009 | 1.09 | 0.93-1.28 | 0.293 | 72.7 | 0.001 |
| Li et al. 2010 | 1.13 | 0.99-1.30 | 0.074 | 63.0 | 0.008 |
| Chen et al. 2011 | 1.11 | 0.93-1.30 | 0.285 | 71.5 | 0.001 |
| Park et al. 2014 | 1.05 | 0.92-1.20 | 0.476 | 55.7 | 0.027 |
| Romieu et al. 2015 | 1.06 | 0.91-1.22 | 0.468 | 65.5 | 0.005 |
| Shin et al. 2015 | 1.11 | 0.96-1.29 | 0.161 | 70.9 | 0.001 |
| Zeinomar et al. 2019 | 1.13 | 0.98-1.30 | 0.083 | 66.3 | 0.004 |

Abbreviations: RR, relative risk; CI, confidence interval

# **Supplementary Table 8. Sensitivity analysis for the association between alcohol consumption and breast cancer recurrences**

| **Study excluded** | **Random effect** | | | **I-squared (%)** | **I-sq. P-value** |
| --- | --- | --- | --- | --- | --- |
|  | **RR** | **95% CI** | **P-value** |  |  |
| Li et al. 2003 | 1.02 | 0.93-1.12 | 0.695 | 49.2 | 0.055 |
| Knight et al. 2008 | 0.99 | 0.92-1.07 | 0.819 | 22.3 | 0.252 |
| Li et al. 2009 | 1.01 | 0.91-1.11 | 0.882 | 46.1 | 0.072 |
| Flatt et al. 2010 | 1.04 | 0.98-1.11 | 0.205 | 0.0 | 0.451 |
| Kwan et al. 2010 | 1.01 | 0.91-1.12 | 0.856 | 46.9 | 0.068 |
| Vrieling et al. 2012 | 1.03 | 0.94-1.14 | 0.512 | 44.5 | 0.082 |
| Holm et al. 2013 | 1.02 | 0.93-1.12 | 0.678 | 48.8 | 0.058 |
| Kwan et al. 2013 | 1.03 | 0.91-1.16 | 0.681 | 49.0 | 0.056 |
| Nechuta et al. 2015 | 1.01 | 0.91-1.12 | 0.880 | 45.9 | 0.074 |

Abbreviations: RR, relative risk; CI, confidence interval

# **Supplementary Table 9. Sensitivity analysis for the association between light alcohol consumption and breast cancer recurrences**

| **Study excluded** | **Random effect** | | | **I-squared (%)** | **I-sq. P-value** |
| --- | --- | --- | --- | --- | --- |
|  | **HR** | **95% CI** | **P-value** |  |  |
| Knight et al. 2009 | 1.02 | 0.93-1.12 | 0.688 | 0.0 | 0.740 |
| Li et al. 2009 | 1.01 | 0.94-1.10 | 0.749 | 0.0 | 0.777 |
| Flatt et al. 2010 | 1.04 | 0.96-1.13 | 0.344 | 0.0 | 0.947 |
| Kwan et al. 2010 | 1.02 | 0.94-1.10 | 0.684 | 0.0 | 0.746 |
| Vrieling et al. 2012 | 1.02 | 0.94-1.10 | 0.655 | 0.0 | 0.741 |
| Holm et al. 2013 | 1.01 | 0.94-1.10 | 0.764 | 0.0 | 0.875 |
| Kwan et al. 2013 | 1.03 | 0.94-1.12 | 0.543 | 0.0 | 0.761 |
| Nechuta et al. 2015 | 1.01 | 0.93-1.10 | 0.881 | 0.0 | 0.809 |

Abbreviations: HR, hazard ratio; CI, confidence interval

# **Supplementary Table 10. Sensitivity analysis for the association between intermediate alcohol consumption and breast cancer recurrences**

| **Study excluded** | **Random effect** | | | **I-squared (%)** | **I-sq. P-value** |
| --- | --- | --- | --- | --- | --- |
|  | **HR** | **95% CI** | **P-value** |  |  |
| Li et al. 2009 | 1.02 | 0.86-1.20 | 0.853 | 0.0 | 0.743 |
| Vrieling et al. 2012 | 1.05 | 0.89-1.23 | 0.592 | 0.0 | 0.981 |
| Kwan et al. 2013 | 1.02 | 0.82-1.26 | 0.876 | 0.0 | 0.725 |
| Nechuta et al. 2015 | 1.01 | 0.84-1.21 | 0.917 | 0.0 | 0.751 |

Abbreviations: HR, hazard ratio; CI, confidence interval

# **Supplementary Table 11. Sensitivity analysis for the association between heavy alcohol consumption and breast cancer recurrences**

| **Study excluded** | **Random effect** | | | **I-squared (%)** | **I-sq. P-value** |
| --- | --- | --- | --- | --- | --- |
|  | **HR** | **95% CI** | **P-value** |  |  |
| Li et al. 2009 | 1.11 | 0.97-1.27 | 0.124 | 25.4 | 0.244 |
| Flatt et al. 2010 | 1.16 | 1.03-1.31 | 0.013 | 0.0 | 0.564 |
| Kwan et al. 2010 | 1.12 | 0.98-1.29 | 0.109 | 27.1 | 0.232 |
| Vrieling et al. 2012 | 1.12 | 0.98-1.29 | 0.088 | 26.8 | 0.234 |
| Holm et al. 2013 | 1.09 | 0.98-1.22 | 0.126 | 0.0 | 0.528 |
| Kwan et al. 2013 | 1.14 | 0.99-1.32 | 0.068 | 21.7 | 0.271 |
| Nechuta et al. 2015 | 1.08 | 0.95-1.22 | 0.255 | 3.2 | 0.396 |

Abbreviations: HR, hazard ratio; CI, confidence interval

# **Supplementary Table 12. Sensitivity analysis for the association between alcohol consumption and breast cancer-specific survival**

| **Study excluded** | **Random effect** | | | **I-squared (%)** | **I-sq. P-value** |
| --- | --- | --- | --- | --- | --- |
|  | **HR** | **95% CI** | **P-value** |  |  |
| Flatt et al. 2010 | 0.95 | 0.89-1.01 | 0.096 | 19.0 | 0.257 |
| Kwan et al. 2010 | 0.93 | 0.86-1.00 | 0.037 | 30.7 | 0.146 |
| Harris et al. 2011 | 0.93 | 0.86-1.00 | 0.054 | 32.9 | 0.127 |
| Vrieling et al. 2012 | 0.93 | 0.86-1.00 | 0.053 | 32.9 | 0.127 |
| Holm et al. 2013 | 0.90 | 0.85-0.96 | 0.002 | 0.0 | 0.687 |
| Kwan et al. 2013 | 0.93 | 0.86-1.00 | 0.059 | 32.6 | 0.130 |
| Newcomb et al. 2013 | 0.92 | 0.85-1.01 | 0.077 | 29.2 | 0.159 |
| Weaver et al. 2013 | 0.93 | 0.87-1.00 | 0.058 | 30.6 | 0.146 |
| Din et al. 2016 | 0.93 | 0.86-1.00 | 0.042 | 32.6 | 0.129 |
| Lowry et al. 2016 | 0.95 | 0.89-1.01 | 0.099 | 21.8 | 0.229 |
| Ma et al. 2019 | 0.95 | 0.88-1.02 | 0.128 | 23.3 | 0.215 |
| Minami et al. 2019 | 0.95 | 0.89-1.01 | 0.101 | 16.4 | 0.283 |
| Zeinomar et al. 2023 | 0.93 | 0.86-1.00 | 0.043 | 32.3 | 0.132 |

Abbreviations: HR, hazard ratio; CI, confidence interval

# **Supplementary Table 13. Sensitivity analysis for the association between light alcohol consumption and breast cancer-specific survival**

| **Study excluded** | **Random effect** | | | **I-squared (%)** | **I-sq. P-value** |
| --- | --- | --- | --- | --- | --- |
|  | **HR** | **95% CI** | **P-value** |  |  |
| Kwan et al. 2010 | 0.92 | 0.84-1.01 | 0.064 | 24.5 | 0.210 |
| Harris et al. 2011 | 0.93 | 0.84-1.02 | 0.115 | 29.1 | 0.169 |
| Vrieling et al. 2012 | 0.91 | 0.86-0.98 | 0.006 | 0.0 | 0.676 |
| Holm et al. 2013 | 0.92 | 0.84-1.01 | 0.084 | 27.7 | 0.181 |
| Kwan et al. 2013 | 0.93 | 0.83-1.03 | 0.144 | 29.1 | 0.168 |
| Newcomb et al. 2013 | 0.93 | 0.83-1.04 | 0.192 | 29.2 | 0.167 |
| Weaver et al. 2013 | 0.93 | 0.85-1.02 | 0.113 | 27.1 | 0.186 |
| Din et al. 2016 | 0.92 | 0.84-1.01 | 0.085 | 27.5 | 0.183 |
| Lowry et al. 2016 | 0.94 | 0.86-1.02 | 0.151 | 25.0 | 0.206 |
| Ma et al. 2019 | 0.94 | 0.86-1.03 | 0.209 | 20.8 | 0.246 |
| Minami et al. 2019 | 0.94 | 0.87-1.01 | 0.080 | 11.5 | 0.335 |
| Zeinomar et al. 2023 | 0.94 | 0.86-1.02 | 0.145 | 25.0 | 0.206 |

Abbreviations: HR, hazard ratio; CI, confidence interval

# **Supplementary Table 14. Sensitivity analysis for the association between intermediate alcohol consumption and breast cancer-specific survival**

| **Study excluded** | **Random effect** | | | **I-squared (%)** | **I-sq. P-value** |
| --- | --- | --- | --- | --- | --- |
|  | **HR** | **95% CI** | **P-value** |  |  |
| Harris et al. 2011 | 0.91 | 0.79-1.04 | 0.173 | 36.5 | 0.138 |
| Vrieling et al. 2012 | 0.91 | 0.80-1.04 | 0.166 | 36.8 | 0.135 |
| Kwan et al. 2013 | 0.88 | 0.78-1.00 | 0.052 | 20.9 | 0.264 |
| Newcomb et al. 2013 | 0.92 | 0.79-1.08 | 0.294 | 35.4 | 0.146 |
| Weaver et al. 2013 | 0.92 | 0.81-1.04 | 0.177 | 34.5 | 0.153 |
| Din et al. 2016 | 0.88 | 0.80-0.97 | 0.011 | 0.0 | 0.590 |
| Lowry et al. 2016 | 0.93 | 0.82-1.06 | 0.259 | 28.0 | 0.205 |
| Ma et al. 2019 | 0.94 | 0.82-1.07 | 0.333 | 27.6 | 0.208 |
| Minami et al. 2019 | 0.92 | 0.81-1.04 | 0.194 | 30.4 | 0.185 |

Abbreviations: HR, hazard ratio; CI, confidence interval

# **Supplementary Table 15. Sensitivity analysis for the association between heavy alcohol consumption and breast cancer-specific survival**

| **Study excluded** | **Random effect** | | | **I-squared (%)** | **I-sq. P-value** |
| --- | --- | --- | --- | --- | --- |
|  | **HR** | **95% CI** | **P-value** |  |  |
| Kwan et al. 2010 | 1.01 | 0.83-1.22 | 0.942 | 58.7 | 0.007 |
| Harris et al. 2011 | 1.03 | 0.84-1.25 | 0.786 | 63.3 | 0.002 |
| Vrieling et al. 2012 | 0.99 | 0.83-1.18 | 0.894 | 53.1 | 0.019 |
| Holm et al. 2013 | 1.05 | 0.86-1.28 | 0.650 | 65.3 | 0.001 |
| Kwan et al. 2013 | 1.09 | 0.88-1.34 | 0.423 | 63.9 | 0.002 |
| Newcomb et al. 2013 | 1.09 | 0.87-1.37 | 0.470 | 63.7 | 0.002 |
| Weaver et al. 2013 | 1.02 | 0.85-1.22 | 0.841 | 60.6 | 0.005 |
| Din et al. 2016 | 1.02 | 0.84-1.24 | 0.839 | 62.2 | 0.003 |
| Lowry et al. 2016 | 1.10 | 0.90-1.33 | 0.367 | 61.7 | 0.004 |
| Ma et al. 2019 | 1.10 | 0.89-1.35 | 0.375 | 61.6 | 0.004 |
| Minami et al. 2019 | 1.07 | 0.88-1.30 | 0.495 | 64.9 | 0.002 |
| Zeinomar et al. 2023 | 1.05 | 0.86-1.29 | 0.628 | 65.4 | 0.001 |

Abbreviations: HR, hazard ratio; CI, confidence interval

# **Supplementary Table 16. Sensitivity analysis for the association between alcohol consumption and overall survival**

| **Study excluded** | **Random effect** | | | **I-squared (%)** | **I-sq. P-value** |
| --- | --- | --- | --- | --- | --- |
|  | **HR** | **95% CI** | **P-value** |  |  |
| Jain et al. 2000 | 0.84 | 0.79-0.89 | <0.001 | 34.0 | 0.097 |
| Reding et al. 2008 | 0.86 | 0.80-0.94 | <0.001 | 80.2 | <0.001 |
| Flatt et al. 2010 | 0.85 | 0.78-0.93 | <0.001 | 82.5 | <0.001 |
| Hellman et al. 2010 | 0.85 | 0.78-0.92 | <0.001 | 82.8 | <0.001 |
| Kwan et al. 2010 | 0.84 | 0.77-0.92 | <0.001 | 83.0 | <0.001 |
| Harris et al. 2011 | 0.85 | 0.78-0.93 | <0.001 | 82.4 | <0.001 |
| Vrieling et al. 2012 | 0.84 | 0.77-0.92 | <0.001 | 83.0 | <0.001 |
| Kwan et al. 2013 | 0.84 | 0.77-0.92 | <0.001 | 82.7 | <0.001 |
| Newcomb et al. 2013 | 0.85 | 0.78-0.93 | <0.001 | 74.6 | <0.001 |
| Weaver et al. 2013 | 0.85 | 0.78-0.92 | <0.001 | 83.0 | <0.001 |
| Nechuta et al. 2015 | 0.84 | 0.77-0.92 | <0.001 | 82.9 | <0.001 |
| Din et al. 2016 | 0.87 | 0.80-0.94 | <0.001 | 79.7 | <0.001 |
| Lowry et al. 2016 | 0.86 | 0.79-0.93 | <0.001 | 80.9 | <0.001 |
| Ma et al. 2019 | 0.84 | 0.77-0.92 | <0.001 | 82.8 | <0.001 |
| Minami et al. 2019 | 0.85 | 0.78-0.93 | <0.001 | 82.5 | <0.001 |
| Zeinomar et al. 2023 | 0.85 | 0.78-0.93 | <0.001 | 82.7 | <0.001 |

Abbreviations: HR, hazard ratio; CI, confidence interval

# **Supplementary Table 17. Sensitivity analysis for the association between light alcohol consumption and overall survival**

| **Study excluded** | **Random effect** | | | **I-squared (%)** | **I-sq. P-value** |
| --- | --- | --- | --- | --- | --- |
|  | **HR** | **95% CI** | **P-value** |  |  |
| Jain et al. 2000 | 0.83 | 0.76-0.90 | <0.001 | 21.0 | 0.237 |
| Reding et al. 2008 | 0.85 | 0.76-0.95 | 0.006 | 76.9 | <0.001 |
| Hellman et al. 2010 | 0.85 | 0.75-0.95 | 0.005 | 77.7 | <0.001 |
| Harris et al. 2011 | 0.85 | 0.75-0.95 | 0.005 | 76.6 | <0.001 |
| Vrieling et al. 2012 | 0.85 | 0.76-0.95 | 0.004 | 77.9 | <0.001 |
| Kwan et al. 2013 | 0.82 | 0.72-0.93 | 0.002 | 78.7 | <0.001 |
| Newcomb et al. 2013 | 0.84 | 0.74-0.95 | 0.006 | 72.0 | <0.001 |
| Weaver et al. 2013 | 0.83 | 0.74-0.94 | 0.002 | 78.7 | <0.001 |
| Nechuta et al. 2015 | 0.83 | 0.74-0.94 | 0.002 | 78.7 | <0.001 |
| Din et al. 2016 | 0.86 | 0.77-0.96 | 0.007 | 74.8 | <0.001 |
| Lowry et al. 2016 | 0.85 | 0.76-0.96 | 0.007 | 75.0 | <0.001 |
| Ma et al. 2019 | 0.84 | 0.74-0.94 | 0.004 | 78.0 | <0.001 |
| Minami et al. 2019 | 0.85 | 0.76-0.95 | 0.005 | 77.4 | <0.001 |

Abbreviations: HR, hazard ratio; CI, confidence interval

# **Supplementary Table 18. Sensitivity analysis for the association between intermediate alcohol consumption and overall survival**

| **Study excluded** | **Random effect** | | | **I-squared (%)** | **I-sq. P-value** |
| --- | --- | --- | --- | --- | --- |
|  | **HR** | **95% CI** | **P-value** |  |  |
| Jain et al. 2000 | 0.83 | 0.76-0.90 | <0.001 | 21.0 | 0.237 |
| Reding et al. 2008 | 0.85 | 0.76-0.95 | 0.006 | 76.9 | <0.001 |
| Hellman et al. 2010 | 0.85 | 0.75-0.95 | 0.005 | 77.7 | <0.001 |
| Harris et al. 2011 | 0.85 | 0.75-0.95 | 0.005 | 76.6 | <0.001 |
| Vrieling et al. 2012 | 0.85 | 0.76-0.95 | 0.004 | 77.9 | <0.001 |
| Kwan et al. 2013 | 0.82 | 0.72-0.93 | 0.002 | 78.7 | <0.001 |
| Newcomb et al. 2013 | 0.84 | 0.74-0.95 | 0.006 | 72.0 | <0.001 |
| Weaver et al. 2013 | 0.83 | 0.74-0.94 | 0.002 | 78.7 | <0.001 |
| Nechuta et al. 2015 | 0.83 | 0.74-0.94 | 0.002 | 78.7 | <0.001 |
| Din et al. 2016 | 0.86 | 0.77-0.96 | 0.007 | 74.8 | <0.001 |
| Lowry et al. 2016 | 0.85 | 0.76-0.96 | 0.007 | 75.0 | <0.001 |
| Ma et al. 2019 | 0.84 | 0.74-0.94 | 0.004 | 78.0 | <0.001 |
| Minami et al. 2019 | 0.85 | 0.76-0.95 | 0.005 | 77.4 | <0.001 |

Abbreviations: HR, hazard ratio; CI, confidence interval

# **Supplementary Table 19. Sensitivity analysis for the association between heavy alcohol consumption and overall survival**

| **Study excluded** | **Random effect** | | | **I-squared (%)** | **I-sq. P-value** |
| --- | --- | --- | --- | --- | --- |
|  | **HR** | **95% CI** | **P-value** |  |  |
| Jain et al. 2000 | 0.92 | 0.82-1.02 | 0.108 | 50.4 | 0.013 |
| Reding et al. 2008 | 0.95 | 0.87-1.05 | 0.315 | 62.0 | 0.001 |
| Flatt et al. 2010 | 0.95 | 0.86-1.05 | 0.291 | 63.3 | <0.001 |
| Hellman et al. 2010 | 0.93 | 0.84-1.03 | 0.151 | 67.8 | <0.001 |
| Kwan et al. 2010 | 0.92 | 0.83-1.02 | 0.104 | 67.1 | <0.001 |
| Harris et al. 2011 | 0.93 | 0.84-1.03 | 0.154 | 67.8 | <0.001 |
| Vrieling et al. 2012 | 0.92 | 0.83-1.01 | 0.094 | 66.6 | <0.001 |
| Kwan et al. 2013 | 0.95 | 0.86-1.05 | 0.289 | 63.5 | <0.001 |
| Newcomb et al. 2013 | 0.93 | 0.83-1.05 | 0.240 | 65.8 | <0.001 |
| Weaver et al. 2013 | 0.92 | 0.84-1.01 | 0.084 | 62.1 | 0.001 |
| Nechuta et al. 2015 | 0.93 | 0.84-1.04 | 0.196 | 67.2 | <0.001 |
| Din et al. 2016 | 0.94 | 0.86-1.04 | 0.223 | 65.8 | <0.001 |
| Lowry et al. 2016 | 0.95 | 0.86-1.05 | 0.276 | 64.3 | <0.001 |
| Ma et al. 2019 | 0.95 | 0.86-1.05 | 0.282 | 64.0 | <0.001 |
| Minami et al. 2019 | 0.94 | 0.85-1.03 | 0.180 | 67.7 | <0.001 |
| Zeinomar et al. 2023 | 0.93 | 0.84-1.03 | 0.148 | 67.8 | <0.001 |

Abbreviations: HR, hazard ratio; CI, confidence interval
